# Reciprocal Analysis of *Francisella novicida* Infections of a *Drosophila melanogaster* Model Reveal Host-Pathogen Conflicts Mediated by Reactive Oxygen and imd-Regulated Innate Immune Response

**DOI:** 10.1371/journal.ppat.1001065

**Published:** 2010-08-26

**Authors:** Madeleine G. Moule, Denise M. Monack, David S. Schneider

**Affiliations:** Department of Microbiology and Immunology, Stanford University School of Medicine, Stanford, California, United States of America; University of Massachusetts Worcester, United States of America

## Abstract

The survival of a bacterial pathogen within a host depends upon its ability to outmaneuver the host immune response. Thus, mutant pathogens provide a useful tool for dissecting host-pathogen relationships, as the strategies the microbe has evolved to counteract immunity reveal a host's immune mechanisms. In this study, we examined the pathogen *Francisella novicida* and identified new bacterial virulence factors that interact with different parts of the *Drosophila melanogaster* innate immune system. We performed a genome-wide screen to identify *F. novicida* genes required for growth and survival within the fly and identified a set of 149 negatively selected mutants. Among these, we identified a class of genes including the transcription factor oxyR, and the DNA repair proteins uvrB, recB, and ruvC that help *F. novicida* resist oxidative stress. We determined that these bacterial genes are virulence factors that allow *F. novicida* to counteract the fly melanization immune response. We then performed a second in vivo screen to identify an additional subset of bacterial genes that interact specifically with the imd signaling pathway. Most of these mutants have decreased resistance to the antimicrobial peptide polymyxin B. Characterization of a mutation in the putative transglutaminase FTN_0869 produced a curious result that could not easily be explained using known *Drosophila* immune responses. By using an unbiased genetic screen, these studies provide a new view of the *Drosophila* immune response from the perspective of a pathogen. We show that two branches of the fly's immunity are important for fighting *F. novicida* infections in a model host: melanization and an imd-regulated immune response, and identify bacterial genes that specifically counteract these host responses. Our work suggests that there may be more to learn about the fly immune system, as not all of the phenotypes we observe can be readily explained by its interactions with known immune responses.

## Author Summary

To infect a host and survive attacks from the host immune system, bacteria express genes that allow them to counteract immune responses. By identifying these genes we can learn how hosts fight infections and how bacteria resist immune attacks. We identified *Francisella novicida* genes that interact with the fruit fly immune system by performing a genetic screen of bacterial mutants. We identified genes that when mutated cause the bacteria to grow poorly within the fly. Many of these genes were shown to help the bacteria survive oxidative stress, providing resistance to an immune response known as melanization. We then identified bacterial genes that interact with another branch of the immune system, the imd pathway, by performing a second screen in imd mutant flies. We identified bacterial mutants that cannot grow in wild-type flies but are rescued in imd mutants, indicating an interaction with this pathway. We followed up one example from this screen and found that mutants in the gene FTN_0869 grow normally inside cells, but cannot grow extracellularly. We found that this was due to being unable to resist previously unexplored aspects of the imd-regulated immune response that help fight off *F. novicida* infections.

## Introduction

The outcome of any infection, whether it be clearance of the infecting pathogen, establishment of a persistent infection, or even death of the host is determined by contributions from both the host and the microbe [1]. To infect a susceptible host microbes express virulence factors, genes that allow the pathogen to invade, colonize, and survive within the host and cause essential pathology. In response, the host initiates an immune response that attempts to clear the pathogen and increase tolerance to the ensuing infection [2]. Consequently, in addition to genes that allow the bacteria to invade host cells and obtain nutrients from its host, a subset of the virulence factors expressed by the microbe must address the need of the bacteria to counteract the host immune response. Exploring this complex interplay between host and pathogen can help us to understand bacterial pathogenesis and define the contributions of the host immune system to bacterial virulence.

One way to explore the host-pathogen relationship is to apply model systems that allow us to dissect the genetics of both sides of the equation simultaneously *in vivo* [3]. In this study, we examine the host-pathogen interactions of *Francisella novicida* with an insect host, *Drosophila melanogaster*, and identify aspects of fly immunity that are most important for fighting *F. novicida* infection as well as the bacterial virulence factors that interact with each of these specific immune responses. *Drosophila* is used as a model of innate immunity because its simplicity and the ease at which it can be used for both forward and reverse genetics has allowed for the identification and characterization of aspects of the innate immune response that are conserved across evolution [4]–[6]. The fly immune response has three effector arms: an inducible antimicrobial peptide (AMP) response, a reactive oxygen response mediated by the activation of the enzyme phenoloxidase and the deposition of the pigment melanin, and a cellular immune response in which foreign invaders are phagocytosed by *Drosophila* hemocytes [7]. The humoral AMP response has been studied extensively and shown to be regulated by two pattern recognition pathways, Toll and imd which have been well-characterized and described, but the regulatory mechanisms of the melanization and cellular immune responses have only recently become the focus of increased interest and have not yet been fully elucidated [8].

Previous studies with pathogenic bacteria in the fly have shown that virulence factors that function in the vertebrate hosts of these pathogens are often required for the microbe to survive in the fly [6]. Recently, this has been shown to be true for the live vaccine strain (LVS) of the virulent pathogen *Francisella tularensis*, a Gram-negative facultative intracellular bacterium that is the causative agent of tularemia [9]. *F. tularensis* can infect a wide range of hosts that includes humans, but is more commonly associated with rabbits and small rodents. Unlike many of the pathogens used in previous fly studies, *F. tularensis* also has a documented arthropod vector phase in its natural life-cycle [10], [11]. While many arthropod-vectored pathogens can only be transmitted by a single specific species, *F. tularensis* is able to infect arthropods ranging from ticks to multiple species of mosquito to biting flies such as deerflies [12]–[14]. This makes the *Drosophila* model system particularly useful for studying both general *F. tularensis* host-pathogen interactions and insect-specific factors.

To date, the fly has primarily been used to dissect the function of known bacterial virulence factors or to demonstrate conservation between fly and vertebrate defenses. Less has been done to use forward genetic approaches in the microbe to identify virulence factors *de novo*. As immunologists we tend concentrate on known signaling pathways that have proven simple to study, are of interest to those working in vertebrates because they are conserved, and those that fit our idea of what the fly's immune response should be. In other words, experiments are driven by the interests of the scientists and not the pathogens. We took a more ecology-based approach and determined, from scratch, what this fly pathogen needs to kill the fly. The advantage of the fly is that it is inexpensive, rapid to use and has extensive genetic tools. The fly could be a useful tool for the identification of new virulence factors rather than a system used to study known factors.

To identify new virulence factors and examine their interactions with the fly immune system, we used the *Francisella novicida* strain U112 to infect flies and performed a genome-wide screen to identify factors required for growth and/or survival within the fly. Many of the genes that we identified are required for resistance to the *Drosophila* innate immune response, particularly to the oxidative stress produced by melanization. This is interesting in particular, because until recently, this pathway had been discarded as having no relevance to microbial infections in the fly [15]–[17]. Our work demonstrates that bacterial mutants can be used as probes of the host immune system to identify what aspects of innate immunity are most important in determining the outcome of an infection.

To identify additional interactions between the host immune system and bacterial virulence factors, we performed a second genetic screen in which we compared the ability of *F. novicida* mutants to grow in wild type flies to flies with an immunity defect known to affect fly survival in *F. tularensis* infections. These flies lack a functional imd signaling pathway, and we anticipated that this would reveal bacterial mechanisms necessary to circumvent the imd-regulated immune response. The imd pathway is primarily described as inducing antimicrobial peptides. Although we identified bacterial genes required to resist antimicrobial peptide killing *in vitro*, we were particularly intrigued to find a subset of genes that when mutated did not appear to change *F. novicida* sensitivity to the antimicrobial peptide we tested yet showed an altered phenotype in imd mutants. This suggests the possibility that there are previously undescribed immune mechanisms that are regulated by the imd pathway.

## Results

### Characterization of *Francisella tularensis* ssp. *novicida* infections of *Drosophila melanogaster*

**Figure 1:**
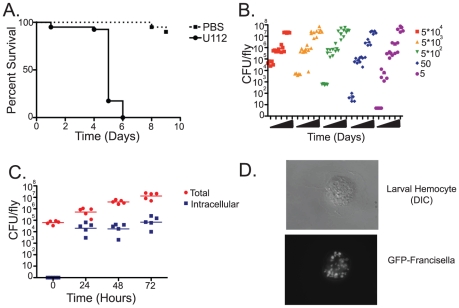
*Francisella novicida* is capable of infecting Drosophila. Wild-type flies were injected with *F. tularensis novicida U112* and survival and growth were monitored over the course of the infection. (A) Survival of wild-type flies following injection of 10^3^ CFUs of *F. novicida*. Median-time-to death (MTD) is approximately five days post infection when incubated at 29°C. Log-rank analysis of the Kaplan-Meyer survival curves showed statistical significance with a P value of <0.0001. Figure S1 provides variance data for these and other survival curves. (B) Growth of *F. novicida* in wild-type flies. Injection of a range of initial doses between 5 and 5*10^4^ CFUs per fly results in bacterial growth to up to approximately 5*10^7^ CFU per fly within 4–5 days post-infection at 29°C. (C) Intracellular and extracellular populations of bacteria within the fly following infection with 5*10^3^ CFU/fly, as determined by survival of bacteria within cells following injection of the non-cell-permeable antibiotic gentamycin. Horizontal lines indicate mean CFU/fly. (D) GFP expressing *F.novicida* within a larval hemocyte.

We infected flies with *F. novicida* strain U112, a wild type strain that causes virulent infections in its natural mouse and rabbit hosts but is not pathogenic to humans. Previous work using the Live Vaccine Strain (LVS) of *F. tularensis* demonstrated that *F. tularensis* grows to high bacterial levels within flies and causes a lethal infection [9]. Infections of the fly with the U112 strain were consistent with this result, although we found the U112 strain to be slightly more virulent in *Drosophila* than the LVS strain, killing the fly with a median time to death (MTD) of 5 days post-infection with 10^3^ CFU (Figure 1A and Figure S1). As few as 5 CFU of *F. novicida* U112 were sufficient to kill the fly, and bacterial growth within the fly was exponential approaching 5×10^7^ CFU per fly before they succumbed to the infection. Regardless of the initial dose, *F. novicida* infections quickly reached the same high levels of bacteria; colony counts in flies receiving a low dose caught up to the 10^8^ fold higher dose within two days (Figure 1B and Figure S2).

In mammalian infections, *F. tularensis* is a facultative intracellular parasite that primarily grows within macrophages [18]. However, due to the extremely high bacterial levels observed within the fly we speculated that a large proportion of the bacteria were growing extracellularly. To test this idea, we performed gentamycin chase assays, infecting flies with *F. novicida* and subsequently injecting them with the non-cell permeable antibiotic gentamycin at various timepoints post infection. This assay kills off extracellular bacteria while leaving bacteria growing within cells intact and allowing us to determine whether the bacteria are growing intracellularly or extracellularly By 24 hours post infection, approximately 1×10^4^ CFU per fly were found to be localized intracellularly. However, the average total population of bacteria in infected flies was at least 1×10^5^ CFU per fly, demonstrating that a significant population of bacteria was located extracellularly. Over the course of the infection the total bacteria population increased to 1×10^7^ CFU/fly, while the intracellular population remained steady, indicating that the extracellular population was responsible for the exponential increase in bacteria seen during fly infections (Figure 1C). This is roughly similar to what has been observed using the LVS strain, although the absolute numbers differ slightly possibly due to the differences in virulence between the two bacterial strains, differences in the host strains or environmental conditions [9].

### Identification of *F. novicida* virulence factors with a Transposon Site Hybridization (TraSH) screen

Having demonstrated that the fly can support *F. novicida* growth we applied this model to the identification of bacterial genes that were important for establishing infection within the fly. Previous work has shown that many *F. tularensis* virulence factors that are required for growth in mammalian models are also essential in insect infections, including the Francisella Pathogenicity Island (FPI) genes iglB, iglC, and iglD and the transcription factor mglA [9], [19]. To expand upon this work, we sought to identify additional bacterial virulence factors and provide an opportunity to discover new biology using a forward genetic approach.

**Figure 2:**
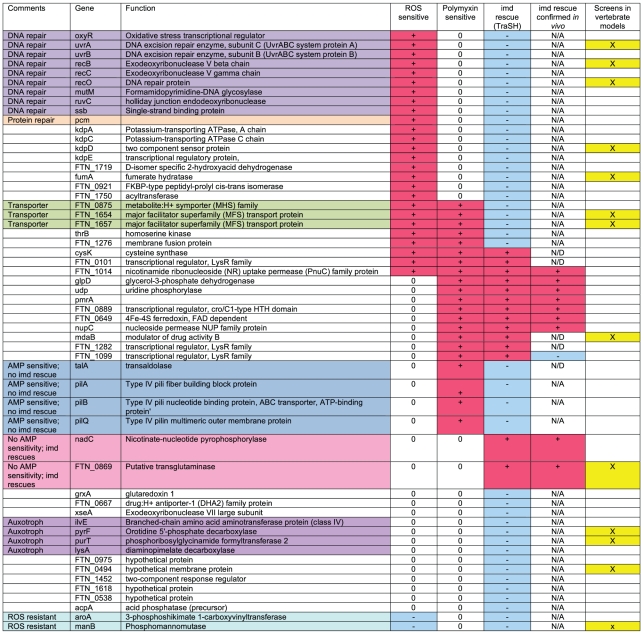
Summary of results of negatively selected mutant phenotypes. Mutants with confirmed attenuated phenotypes by competitive index are categorized by their sensitivity to oxidative stress and polymyxin and phenotype in imd mutant flies. To be considered attenuated, each mutant listed in this table was determined to have competitive indexes that were statistically significantly less than 1 by one sample t-tests with a maximum p value of <0.05. + indicates increased sensitivity, − indicates decreased sensitivity, and 0 indicates no change. N/A indicates that the assay was not applicable to that mutant, and N/D indicates test not done. In the “imd rescue” column, + indicated that the phenotype is rescued in imd mutants, − indicates no rescue. The “screens in vertebrate models” column indicates which mutants were identified in screens for *F.novicida* mutants previously.

Using a transposon insertion library of *F. novicida* mutants we performed an *in vivo* screen for mutants with altered growth rates compared to wild-type bacteria using a Transposon Site Hybridization (TraSH) assay. Briefly, flies were infected with the pooled library and the infection was allowed to proceed for two days, at which point the bacterial populations in each fly were harvested. Genomic DNA was then purified from this population of bacteria and from the original input library that had not been subjected to the stresses found within the fly. RNA was amplified from the site of each transposon insertion and the two populations of RNAs were compared by microarray analysis. We identified mutants representing 149 *F. novicida* genes that were negatively selected with a false discovery rate (FDR) of 5%, indicating that these genes were essential for bacterial growth and survival within the fly (Table S1). 41 of these genes had previously been identified as negatively selected in a similar TraSH analysis performed with the same mutant library in an *in vivo* mouse model; this list includes the known virulence genes iglC, iglD, pdpA, and pdpB, and mglA [20]. In addition, 11 genes from our screen overlapped with data from a negative selection screen performed by *Kraemer et al.* using an inhalation model to observe respiratory infections in the mouse, and an additional 8 overlapped with a signature-tagged mutagenesis screen performed by *Su et al.* that also used an intranasal route of inoculation [21], [22]. The overlap between our TraSH assay and additional *Francisella* genome-wide screens is shown in Table 1 and Figure 2. Interestingly, no genes were identified in all four unique screens, although 7 genes were identified in our fly TraSH and at least two other screens. These genes were the hypothetical proteins FTN_1682 and FTN_1016, the RNA methyltransferase yibK, the ABC transporter yjjK, the amino acid antiporter FTN_0848, iglC and iglD. The degree of overlap between our fly screen and similar mouse screens both supports the hypothesis that our screen in *Drosophila* can be used to identify virulence factors that are conserved between insect and mammalian infections, and also presents the possibility of identifying virulence factors unique to the arthropod vector stage of the *F. novicida* life cycle.

**Table 1:** Overlap between *Drosophila* TraSH screen and screens for *Francisella* virulence factors in a mouse model.

U112 loc	Schu4 loci	Gene Product	Gene	[20]	[21]	[22]
FTN_1683	FTT0028c	drug∶H+ antiporter-1 (DHA1) family protein		x		
FTN_1682	FTT0029c	conserved hypothetical protein		x		x
FTN_1658	FTT0052	Histidyl-tRNA synthetase	hisS	x		
FTN_1657	FTT0053	major facilitator superfamily (MFS) transport protein		x		
FTN_1654	FTT0056c	major facilitator superfamily (MFS) transport protein		x		
FTN_1617	FTT0094c	sensor histidine kinase	qsec	x		
FTN_1582	FFF0134	hypothetical membrane protein		x		
FTN_0210	FTT0295	hypothetical protein		x		
FTN_0217	FTT0303c	L-lactate dehydrogenase	lldD		x	
FTN_0493	FTT0397	5-methylthioadenosine\S-adenosylhomocysteine nucleosidase	mtn	x		
FTN_0494	FTT0398c	hypothetical membrane protein		x		
FTN_0495	FTT0399c	BNR/Asp-box repeat protein		x		
FTN_0554	FTT0463	tRNA/rRNA methyltransferase	yibK		x	x
FTN_0546	FTT0455c	dolichyl-phosphate-mannose-protein mannosyltransferase family protein		x		
FTN_0599	FTT0509c	conserved hypothetical protein		x		
FTN_1066	FTT0615c	metal ion transporter protein		x		
FTN_1038	FTT0645c	conserved hypothetical membrane protein		x		
FTN_1016	FTT0667	hypothetical protein		x	x	
FTN_1220	FTT0790	bacterial sugar transferase family protein		x		
FTN_1214	FTT0797	glycosyl transferase, family 2		x		
FTN_1196	FTT0810c	conserved hypothetical UPF0133 protein	ybaB		x	
FTN_0344	FTT0829c	Aspartate∶alanine antiporter			x	
FTN_0416	FTT0891	lipid A 1-phosphatase	lpxE	x		
FTN_0429	FTT0903	hypothetical protein		x		
FTN_0824	FTT0947c	major facilitator superfamily (MFS) transport protein, pseudogene			x	
FTN_0840	FTT0961	modulator of drug activity B	mdaB		x	
FTN_0848	FTT0968	amino acid antiporter		x		x
FTN_1243	FTT1224c	DNA repair protein recO	recO		x	
FTN_0891	FTT1013	holliday junction DNA helicase, subunit B	ruvB	x		
FTN_1257	FTT1239	membrane protein of unknown function		x		
FTN_1276	FTT1257	membrane fusion protein		x		
FTN_0666	FTT1312c	excinuclease ABC, subunit A	uvrA			x
FTN_0664	FTT1314c	Type IV pili fiber building block protein				x
FTN_1310	FTT1345	conserved hypothetical protein; conserved hypothetical protein	pdpB; pdpB1	x		
FTN_1319	FTT1354	conserved hypothetical protein; conserved hypothetical protein	pdpC	x		
FTN_1321	FTT1356c	intracellular growth locus, subunit D; subunit D	iglD; iglD1	x	x	
FTN_1322	FTT1357c	intracellular growth locus, subunit C; subunit C1	iglC; iglC1	x		x
FTN_1357	FTT1394c	ATP-dependent exoDNAse (exonuclease V) beta subunit	recB	x		
FTN_1417	FTT1447c	Phosphomannomutase	manB	x		
FTN_1501	FTT1490	monovalent cation∶proton antiporter-1		x		
FTN_1439	FTT1531	3-ketoacyl-CoA thiolase	fadA		x	
FTN_1513	FTT1503	site-specific recombinase	xerC	x		
FTN_0337	FTT1600c	fumarate hydratase, class I	fumA			x
FTN_0036	FTT1647c	diyroorotate dehydrogenase	pyrD	x		
FTN_0035	FTT1648c	Orotidine 5-phosphate decarboxylase	pyrF	x		
FTN_1715	FTT1736c	two component sensor protein	kdpD	x		
FTN_1753	FTT1759c	Oxidase-like protein, pseudogene			x	
FTN_1745	FTT1767c	phosphoribosylglycinamide formyltransferase 2	purT	x		
FTN_1762	FTT1782c	ABC transporter, ATP-binding protein	yjjK	x		x

### Confirmation of negatively selected mutants

To confirm the results of the TraSH screen, we tested 65 of the negatively selected mutants individually by competition assay, focusing on mutants that had particularly large decreases in abundance and/or showed homology to bacterial genes that we predicted could play a role in immune evasion or modulation. Transposon insertion mutants of each gene containing a kanamycin resistance cassette were tested to determine their ability to grow in competition with wild-type *F. novicida*. Each mutant was mixed with wild-type bacteria and injected into wild-type *Drosophila* at a 1∶1 ratio. Infected flies were incubated for 48 hours, at which point the bacteria in each fly were plated and the ratio of mutant to wild-type bacterial was determined. A competitive index of 1 represents an equal ratio of mutant to wild-type bacteria, while competitive indexes of less than one indicate that the mutant is attenuated. Mutants that were determined to be statistically significantly less than 1 by one sample t-test in a minimum of three repetitions were considered attenuated and are listed in Figure 2.

**Figure 3:**
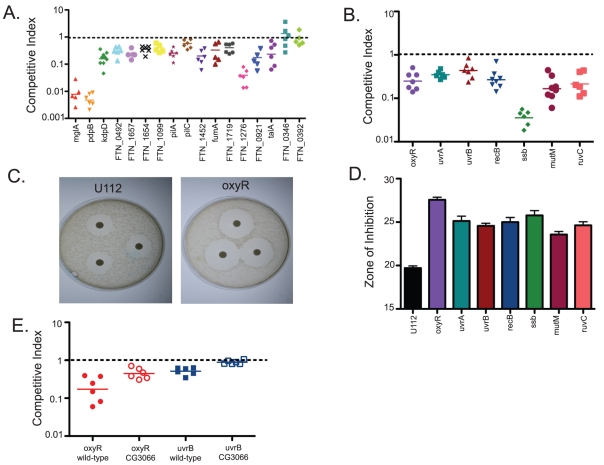
A negative selection screen of *F. novicida* mutants identifies bacterial genes important for bacterial growth and survival within the fly. Transposon Site Hybridization (TraSH) experiments were used to identify bacterial mutants that failed to replicate within *Drosophila* at a rate similar to wild-type bacteria. (A) Candidate mutants were tested individually using competition assays in which each mutant was injected into flies at a 1∶1 ratio with wild type bacteria. Following 2 days of infection, the bacteria from each fly was plated and a competitive index was determined using the ratio of mutant bacteria to wild type bacteria and comparing that to the input ratio. One sample t-tests showed that all mutants shown had competitive indexes significantly different from 1. The P values for each mutant are mglA<0.0001, pdpB<0.0001, kdpD<0.0001, FTN_0494<0.001, FTN_1657<0.0001, FTN_1654<0.0001, FTN_1099 = 0.007, pilA<0.0001, pilC = 0.0035, FTN_1452<0.0001, fumA = 0.0047, FTN_1719 = 0.0003, FTN_1276<0.0001, FTN_0921 = 0.0001, and talA = 0.0016. Two genes that were identified by TraSH but not confirmed as statistically significant, FTN_0346 and FTN_0392 are shown on the far right. Horizontal lines indicate the geometric mean. (B) Mutants of interest identified in the TraSH analysis include bacteria that are impaired in their ability to resist oxidative stress damage, including the transcriptional regulator oxyR and multiple DNA repair pathway genes. One sample t-tests showed that all mutants shown had competitive indexes significantly different from 1. The P values for each mutant are oxyR<0.0001, uvrA<0.0001, uvrB = 0.0004, recB<0.0001, ssb<0.0001, and ruvC<0.0001. Horizontal lines indicate the geometric mean. (C) Disk diffusion assay comparing oxyR to U112 wild-type bacteria demonstrated increased susceptibility to reactive oxygen produced by hydrogen peroxide (D) DNA damage repair mutants are also sensitive to oxidative stress as measured by disk diffusion assay with hydrogen peroxide. Error bars represent standard error. All of the mutants are statistically different than U112 as measured by two-tailed t-tests with P values of oxyR<0.0001, uvrA = 0.0008, uvrB = 0.0002, recB = 0.0008, mutM = 0.0008, ssb = 0.0006, and ruvC = 0.0005 (E) oxyR and uvrB mutants are rescued in CG3066 mutant flies which are unable to produce a melanization response. Both rescues are statistically significant, with P values in a 2-tailed t-test of 0.0165 and 0.0002 respectively. Horizontal lines indicate mean values.

Mutants that were confirmed as negatively selected included kdpA, kdpC, kdpD, and kdpE, components of 2-component regulatory system that responds to turgor pressure, a number of genes known to be regulated by the virulence factor mglA, members of the Major Facilitator Superfamily (MFS) thought to be involved in substrate transport and drug resistance, multiple genes know to be involved in DNA repair, and a number of hypothetical proteins. (Figure 3A and data not shown) 56 of the mutants tested showed attenuated phenotypes by competition assay, with competitive indexes ranging from 0.6-0.007. The results of the competition assays indicated that the microarray data produced by the TraSH assays is useful for predicting negatively selected mutants but was somewhat non-quantitative; the degree of attenuation as measured by microarray analysis did not always correlate with the strength of the phenotype observed by competition assay.

### *F. novicida* mutants demonstrating increased sensitivity to oxidative stress are attenuated in *Drosophila*

One set of negatively selected mutants stood out as particularly interesting because they indicated a bacterial requirement for resistance to oxidative stress within the fly. These mutants included the LysR family transcriptional regulator oxyR. The homologue of this gene in *E. coli* has been shown to sense hydrogen peroxide and induce the transcription of downstream genes that provide protection against oxidative stress [23]. We also identified a number of genes that are essential for repairing damage to DNA such as that caused by reactive oxygen, including uvrA, uvrB, recB, ssb, mutM, and ruvC [24] (Figure 3B). This result is consistent with a screen for attenuated *F. novicida* U112 transposon mutants using an inhalation method of inoculation, which identified the DNA repair proteins recO and recA [21].

The fly's melanization immune response produces reactive oxygen as an effector and thus we hypothesized that these bacterial genes were involved in helping *F. novicida* resist melanization [25], [26]. To test this idea, we first performed *in vitro* disc diffusion assays to determine the sensitivity of each mutant to hydrogen peroxide (H_2_O_2_) and paraquat [27]. The oxyR mutants were extremely sensitive to both H_2_O_2_ and paraquat (Figure 3C (H_2_O_2_) and data not shown (paraquat)). In addition, all of the DNA damage repair mutants showed a significant degree of sensitivity to both reactive oxygen-producing agents (Figure 3D). Taken together, these data suggest that we identified a class of *F. novicida* genes that are essential for wild-type growth and survival within the fly, genes which help the bacteria to resist oxidative stress. Interestingly, 4 DNA repair genes, uvrA, recB, recO, and uvB, were identified in one screen of *Francisella* mutants in mice, suggesting that reactive oxygen species are a threat to the bacteria in mammalian infections as well.

To determine whether melanization is a critical factor limiting the growth of *F. novicida*, we performed competition assays using the oxyR mutant in CG3066 mutant flies. These flies do not induce melanization upon infection [17]. We found that the growth defect of oxyR with respect to wild type bacteria was rescued in non-melanizing CG3066 flies (Figure 3E). This supports the idea that melanization is the reactive oxygen producing immune response for limiting the growth of *F. novicida* in the fly. We therefore took our collection of negatively selected mutants and tested them for sensitivity to reactive oxygen. We found 25 mutants with increased sensitivity to oxidative stress and 2 with decreased sensitivity (Figure 2). Thus we were able to assign functions to these genes based on their behavior in a fly pathogenesis screen. This group of mutants makes up 45 percent of the mutants with attenuated growth phenotypes in the fly, demonstrating that oxidative stress mediated immunity is an important aspect of the fly's defenses against this pathogen and that an important class of *F. novicida* virulence factors in fly infections are genes that help the bacteria to counteract the effects of reactive oxygen.

### Identification of *F. novicida* genes involved in bacterial resistance to imd-regulated innate immune responses

**Figure 4:**
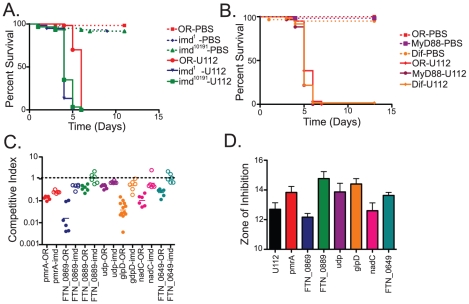
Negative selection screens in Drosophila immunity mutants identify *F. novicida* mutants that help the bacteria resist the imd-regulated host innate immune response. Survival of Toll and imd pathway mutants infected with *F. novicida* at 29°C. (A) Two null alleles of imd, imd^10191^ and imd^1^ backcrossed to OR backgrounds were tested, and both are significantly different from OR flies with log-rank test P values of >0.0001. (B)The Toll pathway is represented by loss of function alleles of two Toll pathway members, Dif and MyD88. Neither are statistically different from wild-type with log-rank test P values of 0.0866 and 0.0582 respectively. (C) Confirmation of mutants identified in the TraSH analysis as attenuated in wild-type flies and rescued in imd mutant flies. All rescues are statistically significant as measured by two-tailed t-tests, with P values of pmrA = 0.0006, FTN_0869 = 0.0001, FTN_0889 = 0.0113, udp = 0.0004, glpD<0.0001, nadC = 0.0465, and FTN_0649 = 0.0030. Horizontal lines indicate the geometric means of the samples. (D) Sensitivity of imd rescue mutants to Polymyxin B as measured by disk diffusion assay. Error bars represent standard error. pmrA, FTN_0889, udp, glpD and FTN_0649 are statistically significantly different from U112 with 2-tailed t-test P values of 0.0299, 0.0041, 0.0495, 0.0065 and 0.0447 respectively. FTN_0869 and nadC are not significantly different than wild-type U112, with P values of 0.1404 and 0.8130.

Having demonstrated that the TraSH method was useful for identifying genes required for growth in the fly and that many of these mutants were involved in counteracting the oxidative stress response of the fly, we decided to expand our study to examine an additional immune pathway and look for similar interactions. We chose to focus on one of the most intensely studied aspects of the fly innate immune response, NF-κB signaling pathways. *Drosophila* has two well-characterized NF-κB pathways (Toll and imd) that are responsible for sensing the presence of microbes and inducing an immune response [7]. Previous work by others demonstrated that the imd pathway is an important component of the *Drosophila* innate immune response to the LVS strain of *F. tularensis*, while the Toll pathway is not [9]. To confirm this for *F. novicida* U112 strain, we infected flies with null mutations in Toll and imd pathway genes. Two separate alleles of imd, imd^1^ and the null allele imd^10191^, as well as mutants lacking the NF-κB homologue Relish showed significantly increased sensitivity to infections with the U112 strain; in contrast, mutants in the Toll pathway components Dif^1^ and dMyD88^C03881^ showed no significant difference compared to wild type (Figure 4A (imd alleles only, relish data not shown) and Figure S1). Therefore, we focused on *F. novicida* genes required to resist the fly's imd mediated response.

To identify such genes, we repeated our TraSH analysis, this time infecting imd mutant flies and compared the results to those found in wild-type flies. We identified 36 genes that appeared to be negatively selected in wild type flies and at least partially rescued in imd mutant flies (Table S2) Subsequent confirmation of these results by competition assay using transposon insertion mutants revealed a subset of 7 mutants that showed reproducible large rescue phenotypes in imd flies (Figure 4C). These genes were the orphan response regulator pmrA, the gene FTN_0889 which is a helix-turn-helix protein and putative transcriptional regulator, glpD which is an anaerobic glycerol-3-phosphate dehydrogenase, the nicotinate-nucleotide pyrophosphorylase nadC, a uridine phosphorylase udp, FTN_0649, a FAD-dependent 4Fe-4S ferrodoxin, and FTN_0869, a hypothetical protein that encodes a putative transglutaminase that is regulated by the virulence factor mglA [28].

Since the imd pathway has been well-characterized as being responsible for inducing antimicrobial peptide (AMP) mRNA levels in response to *F. novicida* and other bacterial pathogens, the simplest explanation for the rescue of these bacterial mutants in flies lacking an intact imd pathway is that they have increased sensitivity to AMPs. This idea is further supported by identification of pmrA in our rescue screen, as pmrA has previously been shown to be sensitive to the antimicrobial peptide polymyxin B *in vitro* [29]. Therefore, we wished to determine whether the other *F. novicida* genes identified in our rescue screen are also sensitive to AMPs. There are 7 families of AMPs in Drosophila, and more than 2 dozen individual AMPs can be expressed during an infection, producing a complex bacteriocidal cocktail. Among the characterized AMPS, four families have been implicated in killing Gram-negative microbes, attacin, cecropin, diptericin, and drosocin. The first three families contain cation rich peptides while drosocin is described as proline rich [7]. It is currently impossible to recreate *in vitro*, the array of AMPs brought to bear on an infecting microbe *in vivo*. We therefore tried testing individual AMPs for their effects on *F. novicida* mutants. Unfortunately, few of these *Drosophila* AMPs are available commercially. We tested a commercial preparation of cecropin and did not detect activity against F.novicida on plates (data not shown). We turned to the cationic antimicrobial peptide polymyxin B, which has been used to model AMP sensitivity in *F. tularensis* in multiple studies [29], [30].

Of the seven genes we confirmed to be rescued in imd mutant flies, we found that five of these genes, pmrA, FTN_0889, glpD, udp, and FTN_0649 were indeed more sensitive to polymyxin B *in vitro*. Suprisingly, mutants in the genes FTN_0869 and nadC did not show any phenotypes in these assays, suggesting that the imd rescue phenotype of these mutants may not be due AMP sensitivity, or at least not to cationic AMP sensitivity (Figure 4D). To determine how common this phenotype was, we expanded our analysis to include the entire set of confirmed attenuated mutants described in Figure 2. We found that twelve of the fourteen *F. novicida* mutants that were rescued in imd mutant flies on arrays showed altered sensitivity to polymyxin B, whereas this was the case with just five of the thirty eight mutants not rescued in an imd mutant (Figure 2). These five mutants were likely exceptions as they also had defects in reactive oxygen sensitivity and in the absence of an imd mediated response would still be sensitive to a melanization response. In our entire set of attenuated mutants, only nadC and FTN_0869 mutants demonstrated the unique phenotype of rescue in an imd mutant fly without showing any increased sensitivity to AMPs, so we chose to focus on one of these genes, the putative transglutaminase FTN_0869 for further analysis.

### FTN_0869 deletion mutants are attenuated in wild-type flies due to clearance of extracellular bacteria by an imd-dependent immune response

The mutation in the gene FTN_0869 was intriguing as it clearly grows better in imd mutants as compared to wild type flies yet the mutant does not demonstrate altered sensitivity to the antimicrobial peptide we tested. The fly produces dozens of AMPs at once and not all of them work by the same mechanism, therefore it is impossible and illogical to eliminate the possibility that a single untested AMP or combination of imd induced AMPs might be responsible for killing *F. novicida*. Regardless, the resistance of FTN_0869 mutants to an AMP raises the question that the imd pathway might be generating an immune response that was not AMP mediated. In addition, the fact that this gene is regulated by the virulence factor mglA which regulates the *F. tularensis* pathogenicity island and many other important virulence factors suggested that it could be particularly important to *F. tularensis* pathogenesis. To determine the extent of the attenuation of FTN_0869 mutants, we examined the growth and survival of these bacteria in individual infections.

**Figure 5:**
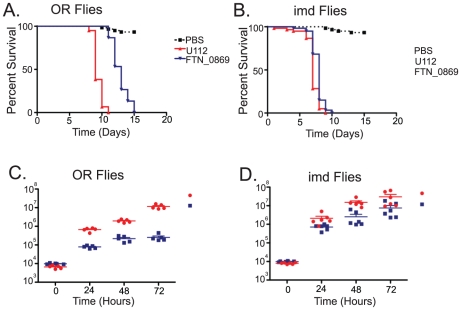
*F. novicida* deletion mutants of a putative transglutaminase are severely attenuated in virulence and growth. (A) Survival of wild-type and FTN_0869 mutant bacteria in wild-type flies at 25°C. FTN_0869 mutants demonstrate significantly lower survival compared to wild-type *F. novicida* with a P value by log-rank analysis of <0.0001. The MTD for U112 is 9 days at 25°C, while the MTD for FTN_0869 is 12 days post infection, with a P value by log-rank analysis of <0.0001. (B) Survival of wild-type and FTN_0869 mutant bacteria in imd mutant flies at 25°C. The FTN_0869 phenotype is now partially rescued as the FTN_0869 mutant and wild-type *Francisella* die with MTDs of 7 and 8 days respectively at 25°C, a 4-fold decrease in the spread between mutant and wild-type survival. (C) Total wild type and FTN_0869 mutant growth in wild type OR flies, showing a growth defect of FTN_0869 mutants. At each timepoint, U112 and the FTN_0869 mutants are significantly different with P values from a 2-tailed t-test of <0.0001. At 24 hours post-infection, U112 has 8.5-fold more CFU/fly than the FTN_0869 mutant. At 48 hours, U112 has 8.9-fold more bacteria, and at 72 hours, U112 infected flies have a full 45-fold more bacteria than the FTN_0869 mutant, with a difference between mutant and wild-type of 5.9*10^5^ at 24 hours, 1.7*10^6^ at 48 hours, and 1.1*10^7^ at 48 hours. Horizontal lines represent the mean CFU/fly at each timepoint. Error bars represent standard error. (D) Growth of wild-type and FTN_0869 mutant bacteria in imd mutant flies, showing rescue of the growth defect. The difference between the mean number of CFUs of wild-type and FTN_0869 has decreased at every timepoint, with only a 2.9-fold increase in wild-type bacteria compared to FTN_0869 bacteria at 24 hours, 6-fold more bacteria in U112 infected flies at 48 hours, and only 4-fold more U112 bacteria than FTN_0869 bacteria at 72 hours. Horizontal lines represent the mean CFU/fly at each timepoint. Error bars represent standard error.

We observed that with a starting dose of 5×10^3^ bacteria the FTN_0869 mutant took significantly longer to kill wild-type flies than did wild-type *F. novicida* (Figure 5A). This phenotype was completely rescued in imd mutant flies, with both FTN_0869 mutants and wild-type bacteria killing the fly with a mean time to death of 7 days, consistent with the sensitivity phenotype observed for imd flies (Figure 5B and Figure S1). In wild-type *Drosophila*, the FTN_0869 mutant did not develop the high bacterial loads found in wild type flies; wild type *F. novicida* can reach titers of 5×10^7^ CFU per fly within 4 days while the FTN_0869 mutant did not grow higher than 5×10^5^ CFU/fly (Figure 5C). Again, this phenotype was abrogated in imd mutant flies, in which both wild type bacteria and FTN_0869 mutants were able to grow to similar high titers. (Figure 5D) This suggested that an imd-regulated immune response was preventing the FTN_0869 mutants from growing as well as wild-type U112 bacteria in the fly. We infer that the decreased bacterial population was responsible for the decreased virulence observed in terms of fly survival.

**Figure 6:**
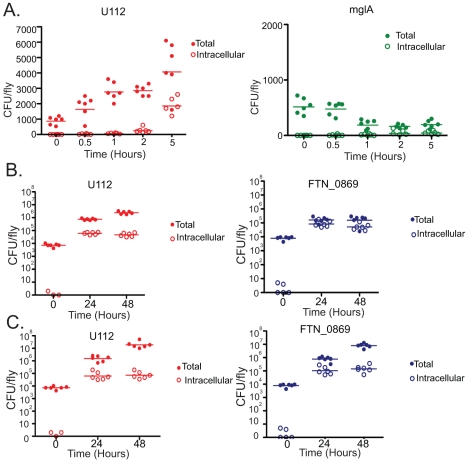
*F. novicida* mglA mutants are unable to survive intracellularly, while FTN_0869 mutants are unable to survive extracellularly. (A) OR flies were treated with gentamycin at 0, 0.5, 1, 2, and 5 hours post infection after incubation at 29°C. Total CFUs per fly and intracellular CFUs as determined following gentamycin treatment for U112 and mglA mutant bacteria. U112 infected flies are represented on the left in red, mglA infection on the right in green. By 30 minutes post infection 1.5% of U122 CFUs are intracellular, at 1 hour post infection this has increased to 2.3%, by 2 hours 9.6% of the bacteria are intracellular, and by 5 hours, 43.4% of U112 is intracellular. In contrast, none of the mglA mutant bacteria is intracellular is intracellular at any timepoint. Horizontal lines represent the mean of all of the data points. (B) Total CFUs in OR wild-type flies and intracellular CFUs and 0, 24, and 48 hours for U112 and FTN_0869 mutant bacteria. U112 infections are graphed on the left in red, FTN_0869 mutant infection on the right in blue. At 24 hours post infection, 8.2% of the U112 CFUs are intracellular, and by 48 hours only 2% is intracellular because the extracellular population is increasing while the intracellular population remains steady. In contrast, at 24 hours post infection 54% of FTN_0869 mutant bacteria is intracellular and 30% is still intracellular at 48 hours, due to the extracellular population failing to increase. Horizontal lines represent the mean. (C) Total CFUs in imd mutant flies and intracellular CFUs and 0, 24, and 48 hours for U112 and FTN_0869 mutant bacteria. U112 infections are graphed on the left in red, FTN_0869 mutant infection on the right in blue. At 24 hours post-infection the percentages of intracellular U112 bacteria in imd flies is similar to those seen in wild-type flies, with 4.6% and 0.4% intracellular at 24 and 48 hours respectively. However, in imd flies the extracellular population of FTN_0868 mutant bacteria is rescued such that only only 15% of the CFUs are intracellular at 24 hours and only 2% is intracellular at 48 hours post-infection. Horizontal lines represent the mean.

The attenuated phenotype of mglA mutants in mouse cells is due to the inability of these mutants to survive and replicate intracellularly [31]. Since FTN_0869 is regulated by mglA, we sought to determine whether the same was true for this mutant. We performed gentamycin chase assays on wild-type U112, mglA mutants, and FTN_0869 mutants. As expected, the mglA mutants showed no bacterial growth within the fly but rather were partially cleared very quickly following injection into the fly, and were completely unable to establish an intracellular population (Figure 6A). This suggests that the small intracellular population may be important, if not essential, for the establishment of a successful infection. In contrast, the FTN_0869 mutants had a robust albeit slightly reduced intracellular population as compared to wild type bacteria, but demonstrated a unique phenotype with little to no extracellular bacteria present in wild-type flies (Figure 6B). By testing sensitivity to gentamycin *in vitro*, we were able to show that this was due to lack of extracellular bacteria rather than an increased sensitivity of the FTN_0869 mutant to gentamycin (Figure S3). Again, loss of the imd pathway in the host animal eliminated this effect; the extracellular population of FTN_0869 mutant bacteria grew to similar levels as wild-type bacteria in imd mutant flies (Figure 6C).

**Figure 7:**
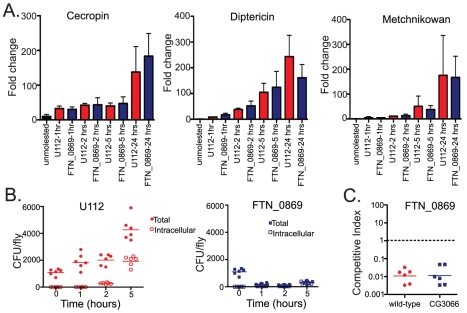
The clearance of extracellular FTN_0869 mutant is not due to altered imd pathway activation, antimicrobial peptide induction, or the *Drosophila* melanization response. (A) Antimicrobial peptide RNA levels for Cecropin, Diptericin, and Metchnikowan as determined by quantitative RT-PCR. Error bars represent standard error. Antimicrobial peptide induction is not significantly different between wild-type and FTN_0869 mutant *F. novicida* infections for any of the AMPs tested at any timepoint. (B) Gentamycin chase experiments for early time points, before the induction of antimicrobial peptides. The kinetics of the clearance of extracellular FTN_0869 mutant bacteria are too rapid to be attributed to antimicrobial peptide induction. At one hour post infection, clearance of FTN_0869 mutants has already begun. While only 2.8% of U112 CFUs are intracellular at 1 hour, 29% of FTN_0869 mutant bacteria is already intracellular presumably because the total number of CFUs present in the fly has reduced from 10^4^ per fly to 2*10^3^ per fly. By two and five hours post-infection at 29°C wild-type bacteria have both extracellular and intracellular populations and have begun to replicate. At 2 hours post infection, the total CFU's of U112 per fly has doubled from 1*10^4^ to 2*10^4^ with 14% of the CFUs intracellular. At 5 hours post-infection, the mean total CFUs per fly is 4*10^4^ with 45% intracellular at 5 hours. In contrast, at 2 hours post infection, 27.6% of the FTN_0869 mutant bacteria are intracellular and the total CFUs per fly remains steady at 10^3^/fly. At 5 hours post infection the bacterial levels have increased slightly to 3*10^3^/fly but the intracellular population has increased to 80% of the total CFUs/fly. Horizontal lines indicate the mean. (C) Competitive indexes of FTN_0869 mutants in wild-type w^1118^ flies and in non-melanizing CG3066 mutant flies two days post-infection at 29°C. There is no statistically significant difference between the competitive indices in wild-type and non-melanizing flies, with a P value of 0.601 by 2-tailed t-test, showing that the FTN_0869 mutants are not rescued in *Drosophila* melanization mutants. Horizontal lines represent the geometric mean of each data set.

This result suggested that the extracellular population of bacteria was unable to persist in the extracellular space of infected flies due to an immune mechanism that is controlled by the imd signaling pathway. Therefore, we were interested in investigating this mutant further to determine what effector arm of the innate immune system was responsible for the clearance of extracellular bacteria. We hypothesized that this clearance could be due to either an increased activation of the imd pathway by the FTN_0869 mutants, AMP activity that we were unable to test *in vitro*, or a novel component of the imd-regulated immune response. To determine if the imd pathway is induced more intensely by the FTN_0869 mutant, to rule out the possibility that this gene is able to downregulate the imd immune response, we measured the induction of imd-regulated AMPS as a readout for imd pathway activation. We used quantitative real-time RT-PCR to monitor the levels of Diptericin, Drosocin, Drosomycin, Attacin, Cecropin and Metchnikowin at 1,2,5 and 24 hours post-infection. We found that only Metchnikowin, Cecropin, and Diptericin were strongly induced in response to *F. novicida* infections and that the transcript levels of each of these highly-induced AMPs peaked at 24 hours post-infection (Figure 7A and data not shown). All of these AMPs were induced to similar levels during infections with either the wild-type or FTN-0869 mutant bacteria, with no statistically significant difference between induction by wild-type or mutant bacteria at any timepoint. This confirms that the imd pathway is indeed activated by *F. novicida* and that the gene FTN_0869 does not have an effect on the induction of the imd pathway.

### Clearance of extracellular FTN_0869 mutants is not dependent on antimicrobial peptides or melanization

We wished to probe the role of AMPs in clearing *F. novicida* further. There are more than 30 antimicrobial peptides in the fly and purified Drosophila AMPs are not readily available and the AMPs are always expressed together during an immune response; as described above, this makes it difficult to directly test the role of AMP activity on *F. novicida* growth in the fly. We therefore tried an indirect approach to test their importance. Recent work in the beetle *Tenebrio molitor* demonstrated that the majority of bacteria injected into the insect is cleared in less than an hour post-infection, much faster than antimicrobial peptides can be upregulated, transcribed, and synthesized [32]. Using this larger insect model, Haine et al. were able to conclusively demonstrate that insect antimicrobial peptide activity is induced slowly, and thus is not responsible for the bulk of the bacterial clearance. The analysis of antimicrobial peptide induction in the fly relies on the analysis of mRNA transcript levels, which are less accurate kinetically than a direct measurement of antimicrobial activity but nevertheless suggest that a slow induction with transcript levels only rising hours after infection and peaking at 6–24 hours post-infection for various AMPs [33]. To determine if the kinetics of extracellular bacterial clearance coincide with AMP induction, we performed gentamycin chase assays at early timepoints post infection. As early as 1 hour post-infection much of the FTN_0869 mutant population had already been cleared from the fly; in contrast the extracellular population of wild-type bacteria did not substantially decrease (Figure 7B). By two hours post-infection, the timepoint at which both wild-type and mutant bacteria had begun to enter cells, the wild-type bacteria now had both intracellular and extracellular populations, while in FTN_0869 mutant infections only the intracellular bacteria had survived clearance. By five hours post infection the extracellular population of U112 wild-type bacteria had begun to increase while the titer of FTN_0869 mutants did not and only the intracellular population remained. This supported the notion that imd-induced AMPs were not responsible for the clearance of extracellular FTN_0869 mutant bacteria, as the bulk of this clearance occurred within an hour post-infection before AMP activity would be upregulated.

We next sought to determine if one of the other effector arms of the fly innate immune system could be causing this phenotype. We first examined the effects of reactive oxygen species on the FTN_0869 mutants. Unlike many of the genes we isolated from our TraSH screen, the FTN_0869 mutants did not show increased sensitivity to reactive oxygen species produced by H_2_O_2_ or paraquat *in vitro* as measured by disk diffusion assay (Figure 2). We next examined the effects of melanization *in vivo* by infecting CG3066 mutant flies with wild type and FTN_0869 mutants. Unlike the oxyR mutants, the FTN_0869 mutants were just as attenuated in CG3066 mutants as they are in the wild-type control (Figure 7C) suggesting that these mutants do not have a defect in resisting reactive oxygen stress and that melanization is not responsible for the FTN_0869 imd rescue phenotype. We concluded that the imd rescue phenotype of FTN_0869 mutants not likely due to cationic antimicrobial peptides or melanization and rather suggested a third category of *F. novicida* interactions with the fly immune system as shown in Figure 2.

## Discussion

Our goal was to dissect the host-pathogen interactions between *Francisella* and *D. melanogaster*. To identify components of this complex system we used a combination of three genetic techniques that enabled us to determine the contributions of both host and microbe to the virulence of the infection. First, we identified bacterial virulence factors necessary to infect the fly using a library of *F. novicida* mutants. Second, we used fly immunity mutants to confirm which host immune pathways were essential for fighting *F. novicida* infections. Finally, we combined these two techniques to identify subsets of bacterial virulence factors that allow the bacteria to counter-respond to specific immune attacks and evade immune clearance. This paper identifies genes from both the pathogen and the host that are components of each of these aspects of the host-pathogen relationship.

To identify bacterial virulence factors, we performed an *in vivo* screen that identified 149 bacteria genes that are important for growth and survival within the fly. 41 of the 149 genes had previously been identified in a similar screen performed with the same bacterial library in the mouse indicating that many bacterial virulence factors are conserved between host species [20]–[22]. Genes that overlap between the *Drosophila* and mouse screens include known virulence factors such as mglA, iglC, and iglD, a number of various transporters, and some of the DNA repair genes we identified as helping *F. novicida* to survive oxidative stress. The remaining genes are unique to our screen performed in the fly model. These genes could either represent *F. novicida* genes that play a role specific to arthropod vectors, demonstrate a stronger phenotype in insects than in mammals, or were not identified in previous screens for experimental reasons.

We note that of the 26 *F.novicida* mutants identified as being sensitive to reactive oxygen, 7 (27%) had been previously identified as being important for virulence in vertebrates. In contrast, of the 16 mutants we found to be polymixin sensitive, only 1 (7%) was identified previously as being important for virulence in vertebrates. The numbers in this study are small enough that differences in representation could be due to chance and therefore future work with more pathogens will be required to confirm the trends seen here; that said, analysis of interactions with the fly's reactive oxygen based immune response seems to be useful predictor of genes that will be of interest to those studying vertebrates. In contrast, analysis of the AMP and imd sensitive mutants is not as robust a tool for identifying mutations that will are relevant in vertebrates.

Secondary screens of these mutants revealed important patterns that shed light onto what particular stresses *F. novicida* encounters within the fly. 25 of the 56 mutants that we confirmed to have reduced competitive indexes compared to wild-type *F. novicida* were also hyper sensitive to oxidative stress *in vitro*. This indicates that preventing or repairing damage caused by reactive oxygen species is an important survival strategy for *F. novicida* in insect infections. Of particular interest among the genes that were sensitive to oxidative stress was the gene oxyR, which has homology to an *E. coli* transcriptional regulator that senses and responds to the presence of hydrogen peroxide by inducing the transcription of catalases and other genes that can counteract oxidative stress ^23^. In addition, our screen identified multiple genes in DNA damage repair pathways that are also sensitive to oxidative stress [24], [25]. We expect that these genes are required to repair damage caused by reactive oxygen species to DNA as has been suggested by *Kraemer et al* [21].

Of the three effector arms that have been characterized in the immune response occurring within the fly's body cavity, the major producer of oxidative stress is the melanization response [15], [17], [26]. Therefore, we speculated that the large number of negatively selected bacterial mutants with oxidative stress sensitivity phenotypes suggested that the melanization response plays a large role in the fly's immune response to *F. novicida*. To test this hypothesis, we performed competition assays with the oxyR mutants in fly mutants that lack a melanization response. As expected, the attenuation of these mutants was rescued in flies that do not melanize and therefore would be expected to not produce toxic oxygen species. This demonstrated that melanization is an essential component of the fly immune response against *F. novicida* and demonstrated that we could use our characterizations of bacterial genes to learn about the fly immune system and understand the host-pathogen relationship. We note that reactive oxygen is a well-established immune effector in the Drosophila gut. Perhaps most microbes encountering the fly will face this immune barrier before encountering internal immune defenses. Thus protection against reactive oxygen is doubly important for fly pathogens [34].

Because microbes must withstand the host immune system to mount a successful infection, we were able to exploit the inherent ability of bacteria to function as metaphorical immunologists to identify which aspects of fly immunity were important to *F. novicida* infections. We next sought to determine if this system could be used in the reverse direction by manipulating the fly immune system to identify which bacterial virulence factors were responsible for interacting with one specific aspect of innate immunity. We did this by performing a second round of our *in vivo* screen for bacterial mutants in an immunocompromised fly. We focused on the imd-regulated humoral immune response, which had previously been identified as important for fighting *F. tularensis* infections [9]. We confirmed that the imd pathway, but not the Toll pathway, was essential in combating *F. novicida* infections, and performed our TraSH assay in imd mutant flies. We identified a subset of bacterial virulence factors that were important for infections of wild-type flies but not imd flies; this imd-regulated immune response has been primarily characterized for its role in the induction of antimicrobial peptides and therefore we tested these mutants for their sensitivity to a cationic, membrane active antimicrobial peptide, polymyxin B [29], [30]. As expected, twelve of the fourteen mutants were sensitive to polymyxin killing *in vitro*, providing another example of how resistance to host immune responses is an important component of bacterial virulence.

We identified 2 bacterial mutants that were not sensitive to polymyxin *in vitro* despite being rescued in imd mutants flies. This phenotype was unexpected as the majority of the literature suggests imd signaling drives antimicrobial peptide production and this is its most important job. We propose three explanations for this phenotype. First, the rescue phenotype could be due to specific sensitivity to additional antimicrobial peptides that were not tested *in vitro*; the bacteria show no sensitivity to polymyxin but could be sensitive to one of the 30 or more AMPs synthesized by flies. Second, the rescue phenotype could be due to the bacterial gene being an inhibitor of the imd pathway; in this case the bacteria would have wild type sensitivity to AMPs but would encounter increased concentrations of them in the fly because the bacteria could not inhibit AMP production. Finally, the rescue phenotype of these bacterial mutants could be due to an aspect of imd-regulated immunity that has not been previously described.

To differentiate between these possibilities, we chose one imd-rescue mutant, the putative transglutaminase FTN_0869 to characterize further in terms of its interactions with the fly immune system. We chose this gene because it had a strongly attenuated phenotype in wild-type flies that was significantly rescued in imd mutants and because it has previously been shown to be regulated by the virulence factor mglA, which is essential for *F. novicida* intracellular growth ^28^. More recently, the homologue of this gene in the extremely virulent Type A *F. tularensis* ssp. *tularensis* strain Shu4 was identified in a transcriptional analysis of genes that are upregulated inside mouse bone marrow-derived macrophages (BMMs) [35]. Interestingly, FTN_0869 deletion mutants in the less virulent U112 strain are unable to replicate in BMMs, but mutants of the homologue of this gene, FTT0989 in the SCHU4 strain did not demonstrate any intracellular replication defect [28], [35]. Further characterization of the FTN_0869 mutants showed that these mutants are attenuated for both lethality to the fly and bacterial growth in an imd-dependent manner. However, unlike its transcriptional regulator mglA, the FTN_0869 mutant is capable of intracellular growth within flies, but is incapable of surviving in the extracellular space. This phenotype is consistent with what is observed in mouse bone marrow-derived macrophages with the virulent Shu4 strain, but not with the phenotype of FTN_0869 deletion mutants in the U112 strain. The reason for this difference is unclear, but it is interesting to note that the ability of the putative transglutaminase deletion mutants to grow intracellularly correlates with its virulence in mammalian and insect hosts.

We found that the phenotype of FTN_0869 deletion mutants in flies is imd-dependent, and used this phenotype to investigate the role of the imd pathway in clearing the extracellular bacteria. With this mutant, we were able to show that the imd rescue phenotype of this particular mutant was not due to modulation of the imd pathway because AMP genes downstream of imd are induced to similar amounts in infections with wild-type and FTN_0869 bacteria. By examining the kinetics of the clearance of extracellular bacteria, we were able to limit the possibility that other imd-induced antimicrobial peptides that we did not test *in vitro* were causing the attenuation of the FTN_0869 mutant. Using non-melanizing mutants, we were able to rule out melanization as the cause of this phenotype, leaving us with the possibility that imd could either be regulating the cellular immune response or an uncharacterized effector arm of fly immunity. Thus the FTN_0869 phenotype suggested a third category of host-pathogen interactions between *F. novicida* and the *Drosophila* innate immune system. Future work with this mutant and other imd-rescue mutants identified in our screen could provide further insight into the biology of the imd-regulated fly immune response.

In summary, reciprocal studies of a pathogen, *F. novicida* and a host, *D. melanogaster*, allowed us to identify genes in the pathogen required to counteract, evade, or resist host immune responses and allow bacterial growth and survival. These studies identified two branches of host immunity that are important for fighting *F. novicida* infections, melanization and imd-regulated immune responses and helped us to understand how the bacteria resists these responses. By identifying the mechanism of one or two bacterial mutants based on their sequence or interaction with fly mutants we developed assays to identify the mechanism of mutants with unknown function. Our work with one of these mutants, FTN_0869, taught us that there is likely more to learn about the fly immune system as there are classes of *F. novicida* mutants that cannot immediately be explained by their interactions with the melanization response or AMPs. Our screen allowed us to pose directed questions and focus our investigations on particular aspects of the host immune system and the microbial strategies to evade this immune response, helping us to identify and characterize components of the host-pathogen relationship.

## Materials and Methods

### Drosophila strains

All experiments were performed in wild-type Oregon Red (OR) flies unless otherwise noted. The imd mutant fly line imd^10191^ is a null allele with a 26-nucleotide deletion at amino acid 179 that results in a frameshift mutation and has been backcrossed onto an OR background. The Toll pathway alleles tested in this study are Dif^1^ which is a complete loss of function mutant and MyD88^C03881^ The CG3066^KG02818^ Sp7 mutant flies are PiggyBack insertion mutants on a w^1118^ background (Bloomington stock number 13494), and w^1118^ flies are used as the wild-type control for these experiments. All experiments were performed on 5–7 day old age-matched male flies that were maintained on dextrose medium at 25°C and 65% humidity in a 12∶12h light dark cycle.

### Bacterial cultures

*Francisella novicida* strain U112 was used for all experiments described in this paper. Bacterial stocks were grown in Tryptic Soy Broth (TSB) supplemented with 0.2% L-cysteine and cultured overnight under aerobic conditions at 37°C. Cultures were grown to an OD_600_ of 1.5–2 and diluted in PBS to OD_600_ 0.005–0.01 for fly infections.

### Fly infections

Flies were anaesthetized with CO_2_ and injected with 50_nL_ of bacteria using a glass needle and a Picospritzer III injector system (Parker Hannifin). Each fly was injected in the ventrolateral surface of the fly abdomen and placed into fresh vials with no more than 20 flies per vial to prevent crowding. Following infection, the flies were incubated at either 25°C or 29°C as noted. Each survival curve was performed using 3 replicates of 20 flies each for a total of 60 flies per condition and each experiment was performed a minimum of three times. The number of dead flies was monitored daily and Kaplan-Meier survival curves were generated using GraphPad Prism software, and statistical analysis was performed using log-rank analysis.

### Determination of bacterial CFUs and gentamycin-chase assays

Individual infected flies were homogenized in 100µL of PBS, serially diluted, and plated onto Mueller-Hinton (MH) agar plates supplemented with 0.025% ferric pyrophosphate (Sigma), 0.1% glucose, 0.025% calf serum (GIBCO), and 0.02% Iso-VitaleX (Benton Dickinson). Plates were incubated overnight and colonies were counted to determine the number of bacterial colony forming units (CFUs) per fly. Statistical significance was determined using unpaired two-tailed *t*-tests. Gentamycin chase experiments were performed as described about except that 50nL of 1mg/mL of gentamycin was injected into each fly 3 hours prior to plating [36].

### TraSH experiments

Three sets of 30 flies were injected with 50nL of the trash library. Each fly received approximately 2*10^5^ CFUs of bacteria, representing approximately 2-fold coverage of the library. The infection was allowed to proceed for two days at 29°C, at which point each fly was homogenized and plated onto MH agar. Plates were incubated at 37°C overnight, and the bacteria were collected and pooled and DNA was collected by phenol-chloroform extraction. Each pool was divided in half and digested with either BfaI or RsaI (NEB) and re-pooled to be used as a template for *in vitro* transcription with a MegaScript T7 Kit (Ambion). The RNA was then purified and used for reverse transcription using a SuperScript III First Strand Synthesis Kit (Invitrogen) and random hexamer primers. The resulting cDNA was labeled with amino-allyl dUTP using Klenow (exo-) enzyme (NEB). The input pool was then labelled with Cy5 and the day 2 pools with Cy3 and hybridized to *Francisella* microarrays as has been previously described [28]. Data was normalized using the Stanford Microarray Database according to the median log_2_ Cy5/Cy3 and filtered using a Cy3 net median intensity of 150 and a regression correlation of >0.6. The dataset was then analyzed using SAM software using a blocked 2-class analysis to identify differences between the input and wild-type or input and imd mutant samples with a false discovery rate of 5% [37].

### Construction of bacterial mutants

Genes that were selected for further analysis were knocked out of *F. novicida* individually to create deletion mutants. Briefly, 500bp of sequence 5′ and 3′ to the gene of interest was amplified from genomic *F. novicida* DNA using Phusion DNA Poylmerase (NEB), and fused onto either side of a kanamycin cassette using a sewing PCR reaction. ^38^ The resulting PCR products were then transformed into chemically competent *F. novicida* U112 as described [28] and the mutants were confirmed by PCR.

### Competition assays

To confirm the bacterial growth attenuation phenotypes, 50nL of a 1∶1 ratio of mutant and wild-type bacteria at an OD_600_ of 0.01 was injected into flies. The infection was allowed to proceed for 2 days at 29°C, following which the flies were homogenized and plated onto MH agar plates with and without 30 mg/mL of kanamycin. Since only the mutant bacteria is capable of growing in kanamycin media, we were able to determine the number of wild-type and mutant bacterial CFUs for each fly by subtracting the number of mutant bacterial CFUs from the total CFUs per fly. A competitive index (CI) was determined using the formula CI = (mutant CFU day 2/wild-type CFU day 2)/(mutant CFU input/wild-type CFU input).

### Disk diffusion assays

To determine the sensitivity of various *F. novicida* mutants to oxidative stress and antimicrobial peptides, disk diffusion assays were performed using protocols adapted from Mohapatra et al. and Bakshi et al. [27], [29]. Briefly, 50µL of overnight cultures of bacteria were plated onto MH agar plates to create a lawn of bacteria. Plates were allowed to dry for 10 minutes, and then 6mm Whatman filter paper disks (Fisher Scientific) were placed onto each plate and inoculated with 10µL of 100mM freshly diluted hydrogen peroxide (Sigma) or 10µL of a 10 mg/mL stock of polymyxin B. Plates were incubated overnight and the diameter of the zone of inhibition was measured for each sample. Three zones were measured for each mutant and each experiment was repeated three times.

### qRT-PCR of antimicrobial peptides

The fold increase of antimicrobial peptide expression following infection by wild-type and FTN_0869 mutant *F. novicida* was determined by isolating RNA from infected flies 6 and 24 hours post-infection by trizol extraction and performing qRT-PCR analysis using an iScript One-Step RT-PCR kit with SYBR Green (Bio-Rad) and a Bio-Rad icycler. The following primer sets were used: cecropin 5′ 5″-tcttcgttttcgtcgctctc-3′, cecropin 3′ 5′-cttgttgagcgattcccagt-3′, drosomycin 5′ 5′-gacttgttcgccctcttcg-3′, drosomycin 3′ 5′-cttgcacacacgacgacag-3′, diptericin 5′ 5′-accgcagtacccactcaatc-3′, diptericin 3′ 5′-cccaagtgctgtccatatcc-3′, attacin 5′ 5′-caatggcagacacaatctgg-3′, attacin 3′ 5′-attcctgggaagttgctgtg-3, drosocin 5′ 5′-ttcaccatcgttttcctgct-3′, drosocin 3′ 5′-agcttgagccaggtgatcct-3′, metchinkowin 5′ 5′-tcttggagcgatttttctgg3′, metchnikowin 3′ 5′aataaattggacccggtcttg-3′, ribosomal protein 15a 5′-tggaccacgaggaggctagg, 3′-gttggttgcatcctcggtga.

## Supporting Information

Figure S1 Alternate plots of survival curves. A second representation of each survival curve presented in Figure 1A, Figure 3A, B, and Figure 4A, B using line graphs showing percent survival rather than Kaplan-Myer survival curves in order to show error bars for each timepoint. Error bars represent standard error.
(1.15 MB EPS)

Figure S2 Dose dependency of *F. novicida* infections of the fly. Doses ranging from 5–50,000 CFU/fly kill the fly with MTDs of 4–6 days post infection. The doses presented in this figure correspond with the doses plotted by CFU in Figure 1B.
(0.54 MB EPS)

Figure S3 *F. novicida* gentimicin sensitivity. Sensitivity of wild-type U112 bacteria and FTN_0869 mutant bacteria to the antibiotic gentamycin as measured by growth in culture overnight at 37°C.
(0.74 MB EPS)

Table S1 Negatively selected mutants identified by TraSH assay in OR flies
(0.04 MB XLS)

Table S2 Bacterial mutants that are attenuated in wild-type flies and rescued in imd mutant flies by in a TraSH assay
(0.02 MB XLS)
